# P02.06. The effect of massage therapy for breast surgery patients

**DOI:** 10.1186/1472-6882-12-S1-P62

**Published:** 2012-06-12

**Authors:** N Drackley, B Bauer

**Affiliations:** 1 Mayo Clinic, Rochester, USA

## Purpose

Massage therapy is used as an adjunct to conventional medical therapy. The goal of this pilot was to evaluate the effect of massage therapy on pain, anxiety, tension and overall wellbeing after breast surgery. The secondary goal was to evaluate the feasibility of a fee-for-service delivery model in a hospital setting.

## Methods

A 3-month pilot was performed offering massage therapy to postoperative breast surgical patients. A certified massage therapist approached patients the day after surgery. Massage duration was determined by discussion between the therapist and patient. Massage was offered at a fee of $1/minute and billed through the institutional billing system. Pain, anxiety and tension levels were documented pre and post massage. Patients completed an anonymous survey regarding their experience.

## Results

Of 64 patients seen during the pilot, 46 patients (72%) elected to have massage. Eighteen patients declined due to: cost (n=2), too ill (n=3), not interested (n=11), dismissed (n=2). The mean duration of therapy was 23 minutes (range 8 – 45). Patients reported massage as very effective for: stress relief (76%), relaxation (82%), pain relief (64%), and somewhat effective for: stress relief (21%), relaxation (18%) and pain relief (31%). In terms of general feelings of wellness, 83% of patients reported massage as very effective. Patient comments regarding the experience were positive. Seventy- nine percent of patients returned post massage surveys and 100% were satisfied (91% very satisfied and 9% satisfied) and 100% would recommend postoperative massage to other surgical patients.

## Conclusion

A massage therapy program can be integrated into a busy surgical unit for postoperative breast surgery patients and may complement surgical therapy to assist with pain, anxiety, relaxation, and overall well-being in the postoperative setting.

